# Can we apply the Mendelian randomization methodology without considering epigenetic effects?

**DOI:** 10.1186/1742-7622-6-3

**Published:** 2009-05-11

**Authors:** Ikechukwu U Ogbuanu, Hongmei Zhang, Wilfried Karmaus

**Affiliations:** 1 Department of Epidemiology and Biostatistics, Norman J Arnold School of Public Health, University of South Carolina, USA

## Abstract

**Introduction:**

Instrumental variable (IV) methods have been used in econometrics for several decades now, but have only recently been introduced into the epidemiologic research frameworks. Similarly, Mendelian randomization studies, which use the IV methodology for analysis and inference in epidemiology, were introduced into the epidemiologist's toolbox only in the last decade.

**Analysis:**

Mendelian randomization studies using instrumental variables (IVs) have the potential to avoid some of the limitations of observational epidemiology (confounding, reverse causality, regression dilution bias) for making causal inferences. Certain limitations of randomized controlled trials, such as problems with generalizability, feasibility and ethics for some exposures, and high costs, also make the use of Mendelian randomization in observational studies attractive. Unlike conventional randomized controlled trials (RCTs), Mendelian randomization studies can be conducted in a representative sample without imposing any exclusion criteria or requiring volunteers to be amenable to random treatment allocation.

Within the last decade, epigenetics has gained recognition as an independent field of study, and appears to be the new direction for future research into the genetics of complex diseases. Although previous articles have addressed some of the limitations of Mendelian randomization (such as the lack of suitable genetic variants, unreliable associations, population stratification, linkage disequilibrium (LD), pleiotropy, developmental canalization, the need for large sample sizes and some potential problems with binary outcomes), none has directly characterized the impact of epigenetics on Mendelian randomization. The possibility of epigenetic effects (non-Mendelian, heritable changes in gene expression not accompanied by alterations in DNA sequence) could alter the core instrumental variable assumptions of Mendelian randomization.

This paper applies conceptual considerations, algebraic derivations and data simulations to question the appropriateness of Mendelian randomization methods when epigenetic modifications are present.

**Conclusion:**

Given an inheritance of gene expression from parents, Mendelian randomization studies not only need to assume a random distribution of alleles in the offspring, but also a random distribution of epigenetic changes (e.g. gene expression) at conception, in order for the core assumptions of the Mendelian randomization methodology to remain valid. As an increasing number of epidemiologists employ Mendelian randomization methods in their research, caution is therefore needed in drawing conclusions from these studies if these assumptions are not met.

## Introduction

The use of genotypes that affect modifiable risk factors to make causal inferences falls under the umbrella of Mendelian Randomization (MR) studies [1,2]. Instrumental variable (IV) methods – the statistical methods that underlie such inferences – have been widely used in econometrics, but not in epidemiology [1,3]. Mendelian randomization refers to the *random *assortment of alleles inherited by offspring from their parents at conception [4,5]. This random assortment of inherited alleles has been likened to a randomized clinical trial (RCT), in which the research subjects are randomly allocated to different genotypes rather than to medical interventions [4]. Mendelian randomization studies include any study that uses genetic variation as a robust proxy for a potential disease risk (which cannot be assessed without biases) for the purpose of making causal inferences about the outcomes of the modifiable exposure [1].

To date, the potential impact of epigenetics on the core assumptions that underlie the use of genes as instrumental variables has not been addressed. This paper opens up this inquiry by assessing the appropriateness of the use of Mendelian randomization as an instrumental variable in the presence of epigenetic modifications of gene expression, and cautions investigators to, at the least, recognize the existence of these limitations. We will delineate the major rationale and the core assumptions of the Mendelian randomization methodology, explore the current understanding of epigenetics, and discuss the methodological challenges that arise from the use of genotypes as instrumental variables for modifiable exposures when epigenetic modifications of gene *expression *are present. The goal of this paper is to emphasize that effect sizes will be biased when the presence of epigenetic phenomena violate the implicit fundamental assumptions in Mendelian randomization studies (and are not compensated for in the analytic models). We will illustrate the occurrence of the epigenetic bias both algebraically and with a data simulation.

## What is currently known

### Mendelian randomization and its shortfalls

Mendelian randomization studies exploit the idea that the genotype only affects the disease status *indirectly *and is assigned *randomly *at meiosis, *independent *of measured and unmeasured (or measured-with-error) confounders [1,5]. These properties define an instrumental variable (IV), which is a variable associated with the outcome only through its robust association with an intermediary variable – the exposure of interest [1]. If the levels but not the function of a potential disease risk factor is determined by a genetic polymorphism, then the *levels *of the risk factor will effectively be assumed to have been randomly assigned at conception. This 'randomization' will potentially obviate the effect of confounding in the test of genotype-disease associations [6].

**Figure 1:**
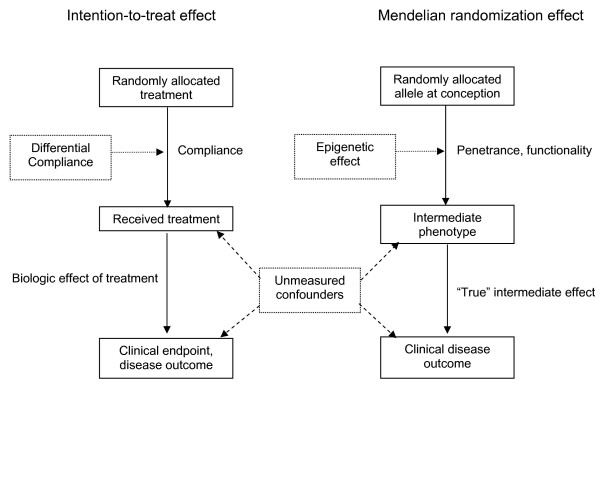
**Conceptual analogies between a randomized controlled trial (left graph) and Mendelian randomization approach (right graph)**. Adapted from Nitsch D, et al. [4].

In order to illustrate the principles of Mendelian randomization, we will use a familiar methodological concept that is well-recognized in randomized controlled trials – the intention-to-treat concept, due to the similarity between the two concepts (i.e. indirect effect and randomized allocation). Randomized controlled trials (RCTs) currently provide the best evidence for potentially therapeutic or prophylactic interventions. RCTs use intention-to-treat (ITT) analysis to assess therapeutic effects [7]. ITT analyses assess *allocated *treatment as the predictor of outcome, and are assumed to be unconfounded because of randomization, irrespective of compliance, adherence and contamination [4,8]. Thus, the ITT effect is the effect of *allocating *a treatment rather than the biologic effect of *received *treatment (Figure 1) [4,7]. Unfortunately, for many types of exposure in epidemiology, it is both impractical and unethical to randomize study participants to different "treatment" arms. Confounder control in observational studies may also be problematic due to incomplete understanding of the relevant confounders in a given situation, or due to inherent measurement errors that arise in the assessment of such confounders [5,9]. Mendelian randomization is a viable strategy for eliminating or reducing residual confounding in observational epidemiological studies [9].

### Core Assumptions Implied in Mendelian Randomization Studies

In order to understand the bias introduced by epigenetic modifications of gene expression, the underlying statistical assumptions in MR studies are outlined next. For this and subsequent sections, let the causal effect of X (intermediate phenotype) on Y be the relationship of primary interest, and let *G *(genotype) be the variable we want to use as the instrumental variable (IV). Also, let U be an unobservable variable that confounds the effect of X on Y.

Three core assumptions characterize an instrumental variable (IV) [1,5].

**Figure 2:**
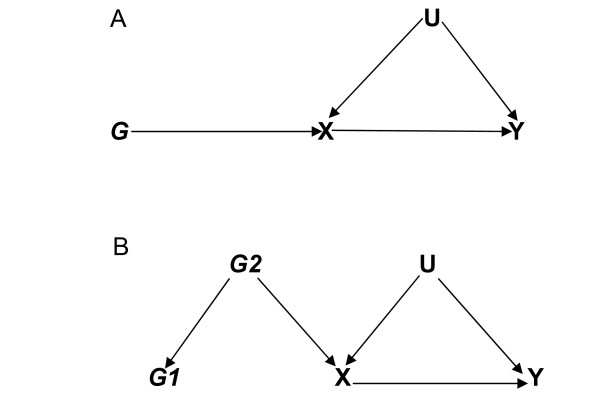
**Directed Acyclic Graph (DAG) specifying the core conditions for an instrumental variable with (2B) and without (2A) the presence of a mediator variable in Linkage Disequilibrium (LD) with the instrumental variable of interest**.

A. *G *is independent of the confounding factors U that confound the association of X and the outcome Y. This assumption accrues from the random allocation of alleles at conception. Any event that alters this allocation could lead to nullification of this assumption (Figure 2A).

B. The instrumental variable *G *is associated with the exposure of interest X (i.e. must not be (marginally) independent of X). The stronger this association, the "better" an instrument *G *is, providing more information on the causal association between X and Y (small standard errors and narrow confidence intervals) [5]. It is important to state at this point that *G *does not need to be causal for X, i.e. to be useful as an IV, *G *does not have to be the "right" gene. The association could instead be due to a mediator variable or through another unobserved variable that affects both *G *and X [5]. This may occur in situations where there is linkage disequilibrium (LD) between *G *(*G1*) and another genotype (*G2*) (Figure 2B).

C. Conditional on X and U, the instrument and the response are independent.

There are several methodological advantages of MR studies. Unlike conventional RCTs, MR studies can be conducted in a representative sample without imposing any exclusion criteria or requiring volunteers to be amenable to random treatment allocation [1]. Secondly, "randomization" in MR studies occurs at conception while RCT studies randomize patients in adulthood. This minimizes biases due to canalization and developmental adaptation [2,4]. Mendelian randomization studies may also counteract some key shortcomings of randomized controlled trials (RCTs), such as high cost, unfeasibility with some exposures, and problems with generalizeability [1,4].

Previous studies have addressed some of the key potential shortfalls of MR studies [2,4,10-14]. These include the paucity of suitable genetic variants, unreliable (genetic) associations, genetic confounding by population stratification, linkage disequilibrium (LD), pleiotropy, functional genomic confounding due to developmental canalization and gene imprinting, the need for large sample sizes and some potential problems with binary outcomes [1]. Other factors that may adversely impact MR studies include selective survival, gene-covariate (environment) interactions and gene-gene interactions. In addition to these potential shortfalls is the potentially distorting effect of epigenetics. This "epigenetic bias" is the focus of this paper.

### What is Epigenetics?

Classical Mendelian inheritance of traits from parents to offspring follows the DNA pairing and transmission patterns as illustrated by the Watson-Crick model of DNA. However, there is an emerging consensus among experts in genetic epidemiology that DNA is not the sole unit of heredity and that genotype and environment are not the only determinants of phenotype [15]. A growing body of evidence suggests that the impact of environmental influences may extend beyond the DNA sequence [16]. The emerging field of epigenetics studies heritable changes in gene expression that occur without directly altering the DNA sequence [17]. Thus, epigenetics is dedicated to the study of non-Mendelian (meiotically and mitotically), heritable changes in gene expression not accompanied by a change in genotype/DNA sequences [14,18-20]. These changes generally involve DNA modification (without change in nucleotide sequence), histone protein modifications, and regulation of gene expression by microRNAs. MicroRNAs (miRNAs) are small (approximately 22 nucleotides long) RNA molecules that may be involved in post-transcriptional control of gene expression [21]. These regulatory mechanisms specify which regions of the genome are active in any given cell at any one time [16,20].

The cells of eukaryotic organisms contain an additional level of information superimposed on the DNA double helix, a nucleoprotein entity known as "chromatin". This "DNA packaging" (or "epigenome") has recently been implicated in the regulation of the complex interactions of the enzymatic processes of replication, transcription, recombination, and DNA repair [16]. The molecular mechanisms of epigenetic modifications include DNA methylation and chromatin structure and histone modifications [19]. Acetylation, methylation, phosphorylation, and ubiquitylation are implicated in *activation*; while methylation, ubiquitylation, sumoylation, deimination and proline isomerization are involved in gene *repression *[18]. However, depending on the location, any given modification is capable of either activation or repression [18]. While acetylation and phosphorylation are thought to be responsible for short-term reversible changes in gene expression, methylation is generally more stable and involved in the long term maintenance of expression status [19]. Inherited changes in the "epigenome" have been postulated as a possible pathway explaining the differences in gene expression seen in individuals with identical "genomes" [22].

A major difference between genetic and epigenetic outcomes is that, while DNA sequence is static, the epigenome is dynamic and changes with cell type, during the cell cycle, in response to biologic signals, and with the environment [16]. Epigenetic effects have been shown to occur not just *in utero*, but over the life course [14]. Certain features of complex diseases that have defied classical Mendelian genetics may be explained, at least in part, by the inheritability, partial stability and reversibility of epigenetic regulation. Gluckman et al. recently proposed that maternally-mediated changes in gene expression may be more relevant in elucidating the etiology of disease in the offspring than the inheritance pattern of the genetic code [23]. In agreement with a possible epigenetic inheritance pattern, a recent study in mice showed that experimentally-induced contact dermatitis in the mother prior to conception resulted in an increased asthma incidence in the offspring [24]. Further strengthening this evidence in humans, Li et al. demonstrated that grandmaternal smoking was associated with an increased risk of asthma in grandchildren, independent of maternal smoking status, suggesting a transmission of epigenetic effects inherited from the grandmother to the grandchild [25].

## Analysis

### Statistical Implications

**Figure 3:**
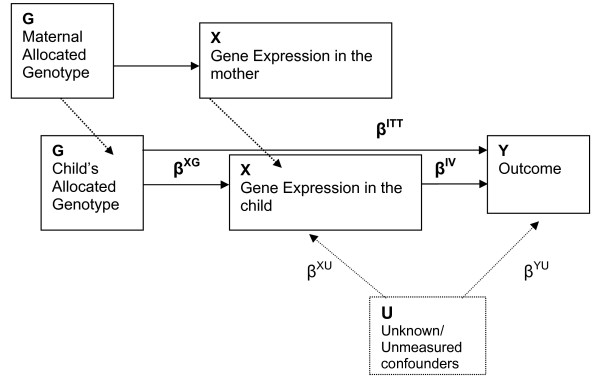
**Three important assumptions in Mendelian Randomization are: a) and b): G associated with X and independent of U.** Thus, the effect of G on X is not affected by U. c) Given X and U, G is independent of Y. Thus, the effect of G on Y can be fully assessed by the effect of G on X and then the effect of X on Y, after adjusting for confounders U; i.e. β^ITT ^= β^XG^β^IV ^as we have shown in Analysis section. (Adapted from Nitsch D, et al. [4]).

Using the RCT analogy for explaining Mendelian randomization in epidemiology, the random allocation *G *affects the outcome (Y) only through received treatment (X), whereas receipt of treatment may be confounded by other variables – known and unknown confounders (U). In the presence of complete blinding, the relation between G and X (i.e. between allocated genotype and intermediate phenotype), β^XG^, is such that G has the same effect on X regardless of compliance; and X has the same effect on Y regardless of the confounding factors (Figure 1). These two steps add up to the (overall) effect of G on Y (Figure 3). As such, the instrumental variable approach can be used to estimate an unconfounded biologic effect on Y of the *received *treatment X, denoted as β^IV ^(Figure 3) [3,26]. It is noteworthy that in Figure 3, MR assumptions imply that the inherited G and X in the child are the same as those of the mother. We thus do not distinguish between the notations for the child's and mother's allocated and received treatments in these algebraic derivations.

Although a more complicated model is possible, to illustrate the IV approach we assume linear relationships among G, X, and Y (i.e. "a one-unit change in G is estimated to result in a β increase in X, and this increase in X in turn is estimated to cause a further increase of β^IV ^in Y, which, multiplied together, gives the total β^ITT ^increase from G to Y" [4]). Following core assumptions A, B, and C (as stated above), Figure 3 can then be modeled as the following, where E denotes expectation of a random variable:

(1)

(2)

(3)

In equations (1) and (3), both β^YU1 ^and β^YU2 ^denote the effect of U on Y. The effect of U is likely to be different when evaluating the contribution of U and G to Y (equation (1)) and the contribution of U and X to Y (equation (3)). Thus, we use different coefficients (β^YU1 ^and β^YU2^) to denote the effects under these two situations. Since U and X are correlated and U is not observable, it is not possible to directly estimate β^IV^. However, since U and G are independent, regressing X on G will provide an estimate of β^XG ^even though U can not be observed. Using the same argument, we can obtain an estimate of β^ITT ^by regressing Y on G. Applying some algebra to the above three equations, specifically by first substituting equation (2) for X in equation (3), and then comparing the effect of G in equation (1) and equation (3), we have:

(4)

which leads to

(5)

Note that in the above derivation, the intercepts are not related to the relationship between β^ITT^, β^XG^, and β^IV^. From Figure 3, estimation of β^IV ^depends heavily on assumptions of compliance, i.e. blinding to allocation (G associated with X and independent of U) and the absence of any other pathway from G to Y (i.e. G being independent of Y given X and U) [4]. An intuitive motivation for equation (5) is that the change in outcome for a unit change in the instrument, β^ITT^, is the product of the change in the exposure for a unit change in the instrument, β^XG^, and the change in the outcome per unit change in exposure, β^IV ^[1]. Estimation of the causal effect β^IV ^by this equation makes use of the variation in Y that is due to the IV, G, and the core assumptions above. Based on our derivation, we can see that this estimate avoids the contaminating effect of the variation in Y due to the confounders of the X-Y association [1].

In Mendelian randomization studies, β^IV ^would be equivalent to the effect of the intermediate phenotype on the outcome (disease) and β^ITT ^to the (direct) genetic effect on disease, while the denominator in equation (5) would capture the observed, or presumed, relation between genetic allocation, G, and its gene product, the intermediate phenotype X [4].

### The Impact of Epigenetics on Mendelian Randomization

**Figure 4:**
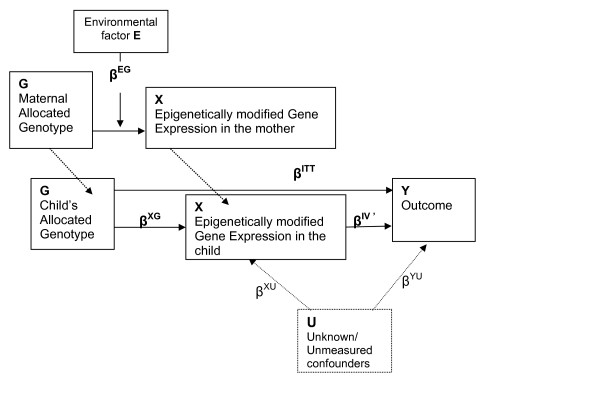
Epigenetic effect present due to inherited altered gene expression (β^XG ^+ β^EG ^= β^XG^*). Compared to Figure 3, when epigenetic effect is present, the core assumptions of MR are violated. Let E denote an environmental factor, which interacts with G at the maternal level. In the presence of this interaction, G and E × G are clearly dependent. Thus, the association between G and X is affected by the E × G interaction term. This violates core assumptions (a) and (b) above. Thus, Mendelian Randomization should be applied with caution if the possibility of epigenetic effects exists. Further, as shown in Analysis section, β^XG^* = β^XG^+ β^EG ^and β^ITT ^= β^XG^*β^IV'^. Therefore, β^ITT ^≠ β^XG^β^IV'^. Thus, when Mendelian Randomization is violated, there is a tendency to contravene the stated relationship between β^ITT^, β^XG^, and β^IV ^as given by equation (5). From the randomized controlled trial analogy, β^ITT ^is the intention-to-treat effect; β^IV^(biologic effect of received treatment); and β^XG ^(the effect of G on X).

Inherited epigenetic effects may alter the ideal gene → "gene product" association discussed above (i.e. in the situation when Mendelian randomization is applicable). From Figure 3 and equations (2) and (3), the less precisely the genotype predicts the gene expression (gene product), the less accurate the derived effect estimate for the causal association between the level of gene expression and the outcome (disease). This necessity for a strong gene → "gene-product" association in order to apply equation (5) requires that there be no substantial biologic co-variation [4]. The impact of epigenetics is due to changes in gene expression (levels of the gene-product) that result in a weaker association. We illustrate this epigenetic effect through an example. In Figure 4, let us assume there is an environmental factor E (e.g. tobacco smoke) taking values 0 (i.e. not exposed) or 1 (i.e. exposed). At the maternal level, factor E interacts with G and this interaction subsequently affects the levels (of expression) of X (i.e. the epigenetic effect which is inherited by the child). Under this assumption, equation (2) is modified as:

(2a)

We use E(X)' to denote the expected gene expression when there is possible gene-environment interaction. Here "E × G" denotes the interaction between the environmental factor E and the genotype G. β^EG ^measures the strength of the interaction and is assumed to be non-zero. We now show that the environmental factor at the maternal level changes the relationship stated in equation (5).

If a subject is not exposed, (E = 0; i.e. no interaction effect), equation 2' is exactly the same as equation 2. In the same vein, the relationship between β^ITT^, β^XG^, and β^IV ^remains as given by equation (5). However, if a subject is exposed to the environmental factor E (i.e. there is an interaction effect), then E = 1. From equation 2', we have

(6)

Let β^XG^* = β^XG^+ β^EG^.

β^XG^* may be greater or less than β^XG ^depending on the predominant mechanism (activation vs. repression, for instance). Note that β^XG^* includes the regular effect of G on X in addition to the effect of gene-environmental interaction that altered the gene expression of the mother's gene. This gene-environment interaction by definition is the epigenetic effect only if it is heritable. As indicated in Figure 4, where such epigenetic effect is inherited by the child, the epigenetically modified gene expression in the child is a result of the G × E interaction inherited from the mother. Thus, the association between X and G in the child includes the effect of G on X and the gene-environment interaction inherited from the mother. (For simplicity in notation, we use the same X and β^XG^* to respectively denote the gene expression in the child and the inherited epigenetic effect, although their values may be different from those at the maternal level).

Further, we use β^IV' ^to denote the effect of gene expression under this assumption, and thus equation (3) becomes:

(3a)

Applying the same algebra as before to equations (1), (3'), and (6), we have:



which gives β^IV' ^= β^ITT^/(β^XG^+ β^EG^) = β^ITT^/β^XG^*, where β^XG^* = β^XG^+ β^EG^.

This new relationship does not agree with that given by equation (5), which is derived based on the MR assumptions. The additional effect (β^EG^) is what we have termed the "epigenetic bias."

It is noteworthy that a gene-environment interaction in parents may lead to an epigenetic effect only when it is inherited by the offspring. Certain gene-environment interactions (in ancestral parents) are thought to be mediated through molecular changes in the epigenome. The *non-random *nature [18] of such alterations may necessitate caution in the interpretation of the estimates from the Mendelian randomization (instrumental variable) analyses. In recognition of the presence of this "epigenetic bias," Bjornsson et al. have suggested that including epigenotypes in models of disease causation might act as a surrogate of *parental *environmental exposure and thus increase the power of epidemiological studies [20].

**Figure 5:**
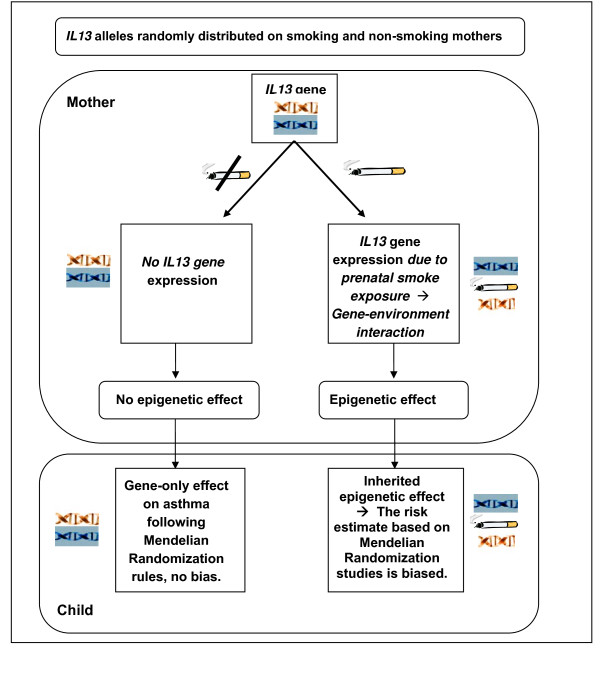
**Schematic representation of possible scenarios of the effects of epigenetics on Mendelian randomization (*IL13*: Inter-leukin-13 gene)**.

Figure 5 illustrates a model of inherited epigenetic effects from mother to child due to environmental influences. Suppose we are studying the effect of *IL13 *gene on asthma, and suppose that maternal tobacco smoke exposure during adolescence resulted in epigenetic changes. Such maternal epigenetic effects could lead to the statistical detection of gene-environment interaction effects in the mother. Further, suppose that at conception, an index child inherited the high-risk *IL13 *("asthma") gene from this former-smoker mother, in addition to inheriting the "epigenetically altered" genotype (epigenome). Although we may not directly find gene-environment interactions in the offspring, the spurious association between the inherited *IL13 *("asthma") gene and the environmental factor (smoking) will nullify core assumption A and adversely affect the use of the *IL13 *gene as an IV. As we alluded to earlier, findings in support of this concept have recently been reported by Li et al. [25] and Sadeghnejad et al. [27]. Li et al. found a multigenerational transmission of asthma across two generations. Their results showed grandmaternal smoking during the mother's fetal period to be associated with a greater risk of asthma in the grandchildren, independent of maternal smoking status. This risk was further heightened when both the grandmother and the mother smoked during pregnancy [25]. In a related finding, Sadeghnejad, et al. [27] demonstrated that offspring who were exposed to maternal smoking during pregnancy and also possessed the risk haplotype of the *IL13 *gene had a higher prevalence of persistent wheezing and asthma in late childhood (evidence of effect modification/statistical interaction). Thus, if the *IL13 *gene is randomly distributed in children, confounding may not be a major concern. On the other hand, distortion of genetic associations assessed using Mendelian randomization may occur due to epigenetic effects. This concept is further illustrated in the data simulation shown in the next section.

Further illustrating conceptually with the randomized clinical trial (RCT) analogy, if an RCT is blinded, intention-to-treat (ITT) cannot be influenced by external factors such as motivation or education (non-differential compliance). However, in a non-blinded trial, participants in the control or treatment arm may choose to alter treatment or compliance (differential compliance, see Figure 1). For instance, control participants may take additional steps to improve their outcome status. This results in an interaction between the allocated treatment and extraneous variables (see Figure 4) and violates the assumptions of ITT. In non-blinded randomized trials, opportunities for extraneous variables to interact with ITT increase with the length of the trial [28]. Similar to this finding in RCTs, in Mendelian randomization studies specific genotypes may interact with a range of extraneous factors and establish epigenetic changes that are inherited by subsequent generations. Thus, the setting of Mendelian randomization is comparable to the setting of a non-blinded randomized clinical trial. Given that the time window between "randomized allocation" of genes at conception and initiation of a MR study is likely to span decades, MR studies are prone to violate the ITT assumption. For instance, if the gene expression of *IL13 *is higher in a child if the mother smoked, then the distribution of the epigenetically modified gene expression is no longer random at birth. Consequently, the child may or may not develop asthma depending on the inception of gene expression in the mother. In essence, since the assumption of MR is to detect an unbiased association between genetic markers and health outcomes due to randomized genes, if studies cannot demonstrate that the gene *expression *is also distributed randomly, the MR model is not justified and will introduce an "epigenetic bias."

### Data Simulation Example

We use simulations to illustrate our findings given in Analysis section. The goal of this simulation is to demonstrate the bias in the estimates of the β coefficients if epigenetic effects transmitted from mother to child are ignored. For this simulation, we assume that a mother smoked in adolescence, stopped smoking as an adult, and then conceived the index study child. Thus, non-epigenetic effects of tobacco smoke exposure *in utero *did not occur. However, gene expression (epigenetic) changes in the mother prior to the index pregnancy were inherited by the child as discussed in Analysis section.

To demonstrate the scenario outlined in Table 1, we assume that we have 10,000 pregnant mothers resulting in 10,000 mother-infant pairs (see attached SAS data and program in additional file 1). For simplicity, we focus on one (asthma) gene (*IL13 *gene) and one single nucleotide polymorphism (SNP) with three possible genotypes (CC, CG and GG). Our simulated data are generated based on the following data scenario:

**Table 1:** Statistical findings of data simulation using log-linear models (scenario outlined in Figure 5)

	Gene → asthma (βITT)		Gene → gene expression of CC (βXG*)		Gene expression of CC → asthma (βIV ')	
	β	SE	Â	SE	β	SE

**Effect of genetic polymorphism on asthma**						
CG/GG (reference)	1					
CC	0.458	0.141				

**Effect of genetic polymorphisms on gene expression:**						
CG/GG (reference)			1			
CC			0.367	0.055		

**Effect of genetic polymorphism, maternal and offspring smoking, and gene-"maternal smoking in adolescence" interaction on gene expression:**						
CG/GG (reference)			1			
CC			0.215	0.086		
Maternal smoking			0.983	0.086		
**"Maternal smoking in adolescence" **× CC-genotype			0.269	0.111		
Offspring smoking			-0.005	0.056		

**Effect of expression of genetic olymorphism on asthma:**						
Gene expression of CC					1.951	0.135

**Effect of genetic polymorphism, maternal and offspring smoking on asthma**						
Gene expression of CC					1.792	0.144
**"Maternal smoking in adolescence"**					0.435	0.145
Offspring smoking					0.166	0.140

# SE = standard error

1) Of 10,000 mothers in the simulated data, 30.5% (n = 3,050) smoked in adolescence, while 69.5% (n = 6,950) did not.

2) The genotypes of mothers who smoked in adolescence and of those who did not were distributed at random: 7.2% of the never-smoking mothers had the GG genotype compared to 9.8% of ex-smoking mothers; 41.7% of never-smoking mothers had the CG genotype compared to 39.3% in ex-smoking mothers; and about 51% in both groups were CC. These percentages were selected using the probabilities that each genotype will be present in a mother.

3) We also assumed that the gene × smoking (G × E) interaction resulted in changes in gene expression in the mother that were inherited by the offspring, leading to asthma manifestation in the offspring.

4) The inheritance of gene expression is assumed to be complete and therefore G and X at the maternal level are consistent with the G and X at the child's level; also paternal genes were assumed not to play any role.

5) Next, we assumed that smoking among the offspring varied with history of maternal smoking: 36.1% of the offspring whose mother smoked in adolescence also smoked, while only 28.2% of the offspring whose mother never smoked also smoked. The overall smoking prevalence for both groups of children was 30.6%. These percentage values were generated from a normal distribution with mean 0.30 and standard deviation 0.10.

6) The data were also simulated such that there was altered gene expression in 25% of the children of ex-smoking mothers compared to 7.4% of the children with never-smoking mothers.

7) Next, 3.6% of the children with ex-smoking mothers had childhood asthma, compared to 1.4% of the children with never-smoking mothers. These percentages were derived from the assumed probabilities of asthma depending on smoking exposure.

8) This simplification is to enable demonstration of the epigenetic effect illustrated in Figure 5. Using the above data scenario, we applied a log-linear model with a dominant effect of the G allele (see SAS program). Hence, the genotypes GG and CG combined served as the reference. We estimated the effects (the values of β) of the CC genotype, maternal smoking, offspring smoking, and the "G × E interaction" for maternal smoking.

As outlined, β's were derived for the following associations:

(1) the effect of the genetic polymorphism on outcome Y (in this case, asthma) estimating β^ITT^;

(2) the effect of the genetic polymorphism on X (in this case, gene expression) estimating β^XG^*

(3) the effect of X (gene expression) on Y (asthma) estimating β^IV^'

The significance level was selected as α = 0.05.

If we ignore the inherited altered gene expression due to maternal smoking in adolescence, we will find a statistically significant crude effect of the CC-genotype on the gene expression (β^XG^* = 0.367, p-value = 0.0001). However, this effect actually includes contributions from both the CC-genotype and from the gene-smoking interaction (G × E interaction) in the mother leading to an epigenetic effect. The contribution from each of these two can be estimated by including a "gene by smoking" interaction term in the model. Inclusion of this term showed a significant gene-smoking interaction effect (β^EG ^= 0.269, p-value= 0.015). Regarding the outcome, asthma (Y), we found a statistically significant effect of the CC-genotype (β^ITT ^= 0.458, p-value < 0.0012).

The above observations imply two important cautionary findings. Firstly, this data simulation demonstrates that the gene effect, even if randomly distributed on the exposure, may be overestimated when the gene × "maternal smoking in adolescence" interaction is ignored. Secondly, we show in this scenario that when the epigenetic effect is ignored, a significant epigenetic effect (gene × smoking interaction) tends to violate the core assumptions of Mendelian randomization. This interaction results in variations in gene-expression and subsequently in increased or reduced risk of the outcome (i.e. an epigenetic effect)

Hence, β^IV' ^= β^ITT^/(β^XG^+ β^EG^) = β^ITT^/β^XG^* as demonstrated earlier, but β^IV' ^is not equal to β^ITT^/β^XG ^as given by equation (4). This is due to the inclusion of gene-smoking interaction, which causes β^XG^* to deviate from β^XG^. As seen in our example, β^ITT ^= 0.458, β^XG^* = 0.367, and β^XG ^= 0.215, so we have β^IV' ^= 0.458/0.367 = 1.248 while β^IV ^= 0.458/0.215 = 2.130. This implies a tendency for β^IV' ^and β^IV^to differ systematically, if evaluated from a study where the gene by "prior maternal smoking" interaction is not taken into account. We refer to this bias as the "epigenetic bias".

## Conclusion

As an increasing number of epidemiologic researchers employ Mendelian randomization in their research, the impact of epigenetics on the core assumptions that drive the use of genotypes as instrumental variables deserves attention. Our work demonstrates that epigenetic bias may distort the effects detected in MR studies.

It is important to differentiate between gene-environment interactions that affect MR studies in the same generation, and epigenetic effects inherited through subsequent generations and that may invalidate MR studies in the second or third generation. Additional tools, such as molecular methods to assess the level of DNA and histone modifications may soon be widely available. Future research in the field of Mendelian randomization needs to collect information on epigenetic changes (e.g. gene expression) of the various genes tested. These additional data would enable adjustment for "epigenetic bias" as described in this paper. Such tools may make it possible to apply certain "correction factors" or to stratify future analyses by "methylation status" (or "expression status"), in the assessment of gene effect [20]. Until such a time, caution is needed in the interpretation of Mendelian randomization studies.

## Abbreviations

LD: Linkage Disequilibrium; DNA: deoxyribonucleic acid; MR: Mendelian Randomization; RCT: randomized controlled trial; ITT: Intention to treat analysis; IV: instrumental variable.

## Competing interests

The authors declare that they have no competing interests.

## Authors' contributions

This paper originated from an epidemiology doctoral class project taught by WK at the University of South Carolina. IUO and WK originated the study idea. IUO wrote the article while WK provided guidance and reviewed initial drafts of the article. HZ offered biostatistical oversight. All authors read and approved the final draft of the manuscript for submission.

## Supplementary Material

Additional File 1 **ogbuanu data**. ogbuanu data simulation program for table 1 Click here for file
